# Recent Advancements in the Surgical Treatment of Brain Tumors

**DOI:** 10.3390/curroncol30090612

**Published:** 2023-09-14

**Authors:** Soichi Oya

**Affiliations:** Department of Neurosurgery, Saitama Medical Center, Saitama Medical University, Saitama 350-8550, Japan; soichi@saitama-med.ac.jp

The surgical removal of brain tumors is essential for improving patient quality of life and survival. Recent advances in medical technology have led to significant advances in brain tumor surgery, the most significant of which are featured in this Special Issue.

1. Improvement in navigation and imaging techniques: Highly accurate navigation and imaging techniques are critical in brain tumor surgery. Recent advances have led to the development of real-time brain imaging and advanced multimodal navigation systems. These changes allow surgeons to accurately locate the tumor and protect the surrounding normal tissue during surgery. This Special Issue also includes a report on the effectiveness of intraoperative advances in navigation, using ultrasonography to address one of the weak points of navigation, a brain shift during surgery.
2. Development of minimally invasive surgery: The development of various optical instruments has also diversified how surgeons view lesions. While traditional brain tumor surgery may require a large craniotomy to access the tumor, recent methods have been established to minimize surgical invasiveness. Some studies in this Special Issue focus on natural orifice surgery via the nasal cavity under an endoscope, which has become a practical approach for treating small- to medium-sized mid-line skull base tumors. In addition, some tumors can be removed via a small craniotomy using an endoscope. These advancements have reduced recovery time and enabled patients’ early reintegration into society. The combination of endo- and exoscope may enhance surgical education for medical students since those in the operating room can share the same three-dimensional images during surgery.
3. Advances in Molecular Targeted Therapy: Recent research has advanced molecular profiling and personalized therapy of brain tumors. This may lead to effective treatment plans based on a patient’s tumor characteristics, potentially improving treatment outcomes. Additionally, accurate prognostication will aid patients in making their own choices. Imaging such as MRI is often insufficient to understanding each patient’s tumor characteristics. In this Special Issue, one study discusses the application of stereotactic biopsy of brainstem lesions for molecular targeted therapy.
4. Laser therapy applications: Recent advances in the laser treatment of brain tumors have made it possible to treat tumors using a noninvasive approach. The effectiveness of laser interstitial thermal therapy for isocitrate dehydrogenase 1 and 2 mutant gliomas is reported in this Special Issue.

As can be seen from the papers featured in this Special Issue, the major change regarding brain tumor surgery over the past 20–30 years is the individualization of treatment. This change has occurred not only in genetic and molecular analysis but also in surgical techniques, which are vital to patient survival and quality of life. The latest findings and technologies have made surgery safer and more effective. With further research and technological advances, treatment options for patients with brain tumors will continue to develop.

